# Relationship between Trunk Muscle Fat Infiltration and Aspiration Pneumonia after Hip Fracture in Older Women: An Exploratory Study

**DOI:** 10.1298/ptr.26-E00009

**Published:** 2026-06-30

**Authors:** Takahiro FUJIMOTO, Shigeharu TANAKA

**Affiliations:** 1 Department of Rehabilitation, Kaneda Hospital, Japan; 2 Department of Rehabilitation, Faculty of Health Sciences, Tokyo Kasei University, Japan

**Keywords:** Hip fracture, Aspiration pneumonia, Trunk muscle, Fat infiltration, Sarcopenia

## Abstract

**Objectives:**

This exploratory study investigated the association between trunk muscle quality assessed at admission, including muscle fat infiltration, and the development of aspiration pneumonia within 12 months after hip fracture in older women.

**Methods:**

This retrospective, single-center study included women aged ≥75 years with hip fracture who underwent abdominal computed tomography at admission. Trunk muscle quality, including muscle fat infiltration and the proportions of normal-attenuation and low-attenuation muscle, was assessed. Participants were classified according to the occurrence of aspiration pneumonia within 12 months after hip fracture, and trunk muscle characteristics were compared between groups.

**Results:**

Women who developed aspiration pneumonia showed a significantly higher intramuscular adipose tissue/total attenuation muscle area index in the erector spinae at admission. In multivariable logistic regression analysis, reduced muscle quality of the erector spinae was associated with the subsequent development of aspiration pneumonia (odds ratio, 1.29; 95% confidence interval, 1.05–1.60).

**Conclusions:**

Fat infiltration in the erector spinae muscle may be associated with the subsequent development of aspiration pneumonia in older women with hip fracture. Trunk muscle quality at admission may reflect underlying vulnerability. However, these findings should be interpreted with caution given the exploratory design, small sample size, and potential residual confounding; no causal relationship can be established.

## Introduction

Hip fracture is a major cause of morbidity and mortality in older adults, with a 1-year mortality rate of approximately 10%–20% in Japan^1,2)^. Therefore, early identification of risk factors for adverse outcomes is important for guiding physical therapy interventions and improving functional recovery.

Aspiration pneumonia is a frequent and serious complication in patients with hip fracture and has been associated with prolonged hospitalization, rehospitalization, and increased mortality risk^3–5)^. Dysphagia plays a central role in the development of aspiration pneumonia. Patients with hip fracture are particularly susceptible to sarcopenia due to hospitalization, reduced physical activity, and inflammatory responses, which contribute to declines in both muscle mass and muscle quality^6)^. Sarcopenia affects not only appendicular muscles but also swallowing-related and respiratory-support muscles, thereby increasing vulnerability to dysphagia^7)^.

Several studies have indicated that swallowing function is influenced not only by local swallowing muscles but also by the functional and structural status of trunk muscles involved in postural control and respiratory regulation. Previous studies suggest that trunk muscle function is associated with swallowing performance, indicating that trunk muscle impairment may contribute to aspiration risk^8,9)^. These findings suggest that vulnerability of the trunk muscles may contribute to impaired swallowing function and an increased risk of aspiration.

By contrast, muscle quality, rather than muscle quantity, has emerged as an important determinant of functional decline and adverse outcomes. Myosteatosis, a marker of reduced muscle quality, reflects fatty infiltration within skeletal muscle and has been associated with adverse outcomes independent of muscle mass^10–12)^.

Trunk muscles are essential for postural maintenance, respiratory assistance, and trunk stability, and deterioration in their muscle quality may reflect systemic vulnerability as well as impairments in swallowing-related functions. Although myosteatosis is highly prevalent in patients with hip fracture, its clinical significance and association with aspiration pneumonia have yet to be fully clarified. Therefore, this study aimed to explore whether reduced trunk muscle quality, assessed using abdominal computed tomography (CT) images obtained at baseline, is associated with the development of aspiration pneumonia within 12 months after hip fracture. This study was conducted as an exploratory investigation to generate hypotheses for future risk stratification and early intervention strategies.

## Methods

### Study design and participants

This single-center, retrospective cohort study was conducted in Maniwa City, Japan, between April 2015 and December 2016. The inclusion criteria were as follows: (1) women admitted to the hospital with hip fractures and with available follow-up data 12 months after injury, and (2) women aged ≥75 years who underwent abdominal CT scanning as part of routine clinical care for diagnostic purposes based on clinical indications. This age threshold was selected based on the proposal by the Japan Geriatrics Society to redefine older adults as individuals aged ≥75 years^13)^. Women were selected because they are more prone to disuse-related muscle atrophy and show age-related increases in intramuscular adipose tissue (IMAT) within the trunk muscles^14,15)^. Pre-fracture physical activity status was determined based on the degree of independence in daily living among disabled older adults, as defined by the Ministry of Health and Welfare in Japan^16)^. The exclusion criteria were: (1) muscle changes attributable to non–age-related causes of sarcopenia, (2) neuromuscular conditions (e.g., Parkinson’s disease) or cerebrovascular disease, (3) active cancer treatment or a history of cancer, and (4) incomplete clinical or imaging data. All eligible women during the study period were included. No deaths occurred during the 12-month follow-up period; therefore, all participants were included in the final analysis.

### Basic characteristics

Basic demographic and clinical information were collected from electronic medical records, including age, height, weight, body mass index (BMI), serum albumin level, length of hospital stay, surgical procedure (bipolar hip prosthesis or open reduction and internal fixation), discharge destination (home or institutional care), and dietary form at discharge (regular, soft, or chopped diet).

### Physical activity

Pre-admission physical activity during the 6 months prior to hospitalization was evaluated using the degree of independence in daily living, as defined by the Ministry of Health and Welfare in Japan^16)^. The scale categorizes older adults into 3 functional levels. Individuals classified as Independent (Rank J) were able to perform daily activities independently and go outside without assistance. Those classified as Semi-bedridden (Rank A) were mostly independent within the home but were unable to go outdoors without help. Individuals classified as Bedridden (Rank B) required assistance for most indoor activities.

### Muscle measurements

CT images used for muscle assessment were obtained at the time of hospital admission and were defined as baseline measurements. Subsequent development of aspiration pneumonia was assessed during the 12-month follow-up period after the hip fracture. Muscle quality was assessed on a single axial CT slice at the superior margin of the third lumbar vertebra (L3). Regions of interest (ROI) for the rectus abdominis, abdominal oblique muscles, psoas major, quadratus lumborum, multifidus, and erector spinae were manually traced by a trained physical therapist using ImageJ (National Institutes of Health, Bethesda, MD, USA). Muscle components were quantified using established Hounsfield unit (HU) thresholds: IMAT was defined as −190 to −30 HU, low-attenuation muscle area (LAMA) as −29 to +29 HU, and normal-attenuation muscle area (NAMA) as +30 to +150 HU. Cross-sectional areas were automatically calculated from pixel counts, and total skeletal muscle area (SMA) and total attenuation muscle area (TAMA) were derived accordingly.

Sarcopenia and myosteatosis were assessed using female cutoff values reported by Kim et al.^17,18)^: SMA/BMI index <3.46 and NAMA/TAMA index <65.1. Based on these criteria, NAMA/TAMA, LAMA/TAMA, and IMAT/TAMA indices were calculated for both total trunk muscle area and individual muscles. A representative CT image illustrating ROI placement is shown in Figure 1.

**Fig. 1.**
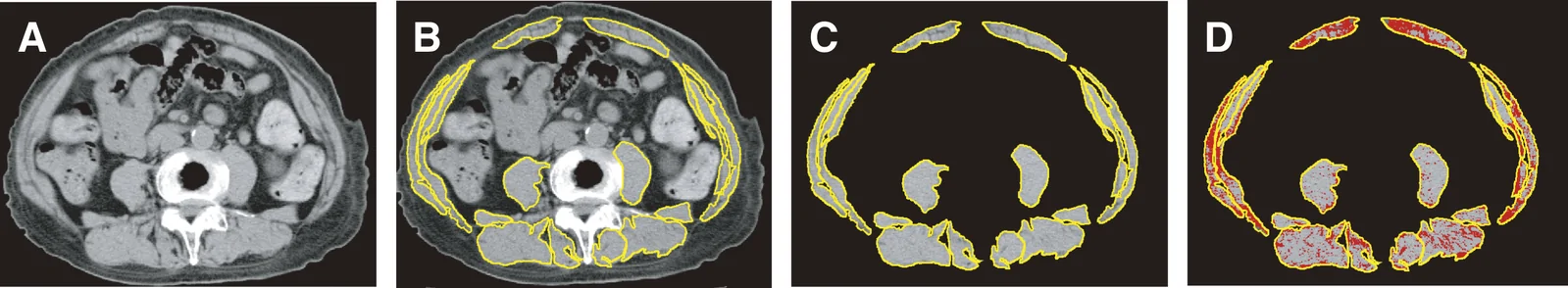
Representative CT image and ROI segmentation at the L3 level. (**A**) Original axial CT image at the L3. (**B**) Manual delineation of trunk muscle ROIs, including the erector spinae, multifidus, psoas major, quadratus lumborum, rectus abdominis, and abdominal oblique muscles. The yellow outlines represent the manually traced ROIs of the target muscles. (**C**) Extracted muscle area after ROI segmentation. (**D**) Illustrative example of HU-based muscle component extraction within the segmented ROI. Red areas indicate voxels within the ROI that meet the predefined HU criteria for the selected muscle component. Muscle components were classified based on predefined HU thresholds as follows: IMAT (−190 to −30 HU), LAMA (−29 to +29 HU), and NAMA (+30 to +150 HU). All image analyses were performed using ImageJ software. CT, computed tomography; ROI, regions of interest; L3, third lumbar vertebra; HU, Hounsfield unit; IMAT, intramuscular adipose tissue; LAMA, low-attenuation muscle area; NAMA, normal-attenuation muscle area

### Outcome measures

The primary outcome was the development of aspiration pneumonia within 12 months following the hip fracture. Aspiration pneumonia was diagnosed by the attending physicians based on newly appearing pulmonary infiltrates on chest radiography or CT, together with clinical symptoms such as fever, productive cough, or elevated inflammatory markers. Aspiration was considered when choking episodes, dysphagia, or clinically suspected aspiration events were documented. Outcome data were obtained from electronic medical records. All women were followed up routinely on an outpatient basis within 12 months post-fracture, and any hospitalization for aspiration pneumonia during this period was reviewed. Women with documented aspiration pneumonia were classified into the aspiration pneumonia group, whereas those without any medical record indicating a pneumonia diagnosis or treatment were classified as the non-aspiration pneumonia group. All outcome events occurred after the baseline CT assessment. During hospitalization, standardized swallowing assessments were not routinely performed. Patients were clinically monitored for signs of dysphagia, including choking or coughing during meals, wet hoarseness, oral residue, and suspected aspiration events; nevertheless, dysphagia could not be systematically evaluated in terms of presence or severity, and these observations may have been insufficient to detect mild or subclinical swallowing dysfunction.

### Bias and missing data

To minimize information bias, all measurements were performed by a single trained assessor blinded to clinical outcomes. Only patients with complete clinical and imaging data were included; therefore, no missing data were imputed.

### Statistical analysis

A priori sample size calculation was not performed because this was an exploratory, hypothesis-generating study.

Continuous variables are presented as median (interquartile range). Differences between groups were examined using the Mann–Whitney U test. Categorical variables were compared using Fisher’s exact test. Dietary form at discharge was compared using the Freeman–Halton extension of Fisher’s exact test. Differences in each muscle-specific index (NAMA/TAMA, LAMA/TAMA, and IMAT/TAMA) between groups were assessed using the Mann–Whitney U test.

To explore factors associated with aspiration pneumonia, logistic regression analyses were performed separately for each trunk muscle. For each muscle, 3 models were constructed using NAMA/TAMA, LAMA/TAMA, and IMAT/TAMA indices as independent variables; to avoid multicollinearity, these indices were analyzed in separate models. Each trunk muscle was analyzed independently; no multivariable model including multiple muscles was constructed.

Given the limited number of events (n = 14), the number of explanatory variables was restricted to minimize overfitting, based on commonly accepted recommendations regarding events per variable. All modeling strategies, including restriction of the number of explanatory variables and criteria for covariate inclusion, were predefined based on methodological considerations for small-sample regression analyses. Accordingly, each model included 1 muscle-specific index. BMI was included as an additional covariate only in models where the events-per-variable ratio remained acceptable (≥10 events per variable), to ensure model stability. In models where this criterion could not be met, BMI was not included. No stepwise variable selection procedures were applied. This modeling approach was selected to reduce the risk of overfitting and enhance model stability given the limited number of events.

Odds ratios (ORs), 95% confidence intervals (CIs), and 2-tailed p-values were calculated. Firth’s bias-reduced logistic regression was performed as a sensitivity analysis to assess the robustness of the findings.

Several clinically relevant variables (e.g., swallowing function and nutritional status) were considered as potential confounders; however, they were not included due to limited availability in this retrospective dataset and the small number of events, which restricted model complexity and may have resulted in residual confounding. Dietary form at discharge was also not included in the regression models, as it may represent an indirect and potentially misclassified surrogate of swallowing function.

All analyses were performed using EZR (version 1.32; Jichi Medical University, Saitama, Japan), with a significance level set at 5%. Given the exploratory nature of this study and the multiple comparisons performed, the findings should be interpreted with caution. These analyses were intended to explore muscle-specific associations rather than to establish causal relationships.

### Ethical considerations

This study was approved by the Ethics Committee of Kaneda Hospital, Social Medical Corporation (approval number: 0503), and was conducted in accordance with the Declaration of Helsinki. Written informed consent for the use of clinical data and follow-up information was obtained from all participants at the time of hospitalization as part of routine clinical procedures. In addition, an opt-out option was provided.

## Results

The study included 31 women, of whom 17 did not develop aspiration pneumonia and 14 did. Aspiration pneumonia occurred at a median of 247.0 (171.0–295.3) days after the hip fracture. All participants were followed for up to 12 months after hip fracture. Women without aspiration pneumonia had higher BMI and serum albumin levels than those with aspiration pneumonia (Table 1). There was no significant difference in length of hospital stay or surgical procedure between the 2 groups (length of hospital stay: p = 0.590; Fisher’s exact test for surgical procedure, p = 1.00; OR: 1.02, 95% CI: 0.23–4.45). Discharge destination did not differ significantly between the groups (13/17 [76.5%] vs. 11/14 [78.6%] discharged home; Fisher’s exact test, p = 1.00; OR: 0.89, 95% CI: 0.16–4.85). Sarcopenia was markedly more prevalent in women who developed aspiration pneumonia, with 12 of 14 patients affected, compared with only 2 of 17 patients in those without aspiration pneumonia (p <0.001). Because all women met the criteria for myosteatosis, comparisons based on its presence or absence were not possible, and muscle quality was analyzed using continuous CT-based indices. Pre-fracture functional status did not differ significantly between the groups (Table 1). Dietary form at discharge did not differ significantly between groups (Fisher’s exact test, p = 0.45). In the without aspiration pneumonia group, 7 patients received a regular diet and 10 received a soft diet, whereas in the with aspiration pneumonia group, 6 received a regular diet, 7 a soft diet, and 1 a chopped diet.

**Table 1.** Baseline characteristics of women with and without aspiration pneumonia

	Without aspiration pneumonia (n = 17)	With aspiration pneumonia (n = 14)	p-Value
Age	86.0 (83.0–87.0)	84.0 (82.3–88.0)	0.675
Height (cm)	147.0 (142.2–154.0)	145.0 (141.3–149.4)	0.735
Weight (kg)	45.1 (37.6–50.8)	40.1 (36.0–41.7)	0.099
BMI (kg/m^2^)	20.7 (19.0–23.7)	18.6 (17.2–19.8)	0.047
Albumin (g/dL)	3.7 (3.5–4.1)	2.9 (2.7–3.2)	0.001
Length of hospital stay (days)	43.0 (34.0–48.0)	49.5 (41.5–53.5)	0.590
Surgical procedure, n (%)			1.000
Bipolar hip prosthesis	6 (35.3)	5 (35.7)	—
Open reduction and internal fixation	11 (64.7)	9 (64.3)	—
Pre-fracture functional status, n (%)			0.081
Independent to semi-independent (Rank J/A)	17 (100)	11 (78.6)	—
Rank J	2 (11.8)	0 (0)	—
Rank A	15 (88.2)	11 (78.6)	—
Dependent (Rank B)	0 (0)	3 (21.4)	—

Values are presented as median (interquartile range) or number (%). p-Values were calculated using the Mann–Whitney U test for continuous variables and Fisher’s exact test for categorical variables. Functional status was classified according to the Japanese Ministry of Health and Welfare scale: Rank J (independent), Rank A (semi-independent), and Rank B (dependent).

n, number; BMI, body mass index

Trunk muscle measurements are summarized in Table 2. Women with aspiration pneumonia showed significantly higher IMAT/TAMA and LAMA/TAMA indices and lower NAMA/TAMA indices compared with those without aspiration pneumonia.

**Table 2.** Comparison of sarcopenia, myosteatosis, and trunk muscle composition between women with and without aspiration pneumonia

	Without aspiration Pneumonia (n = 17)	With aspiration Pneumonia (n = 14)	p-Value
Total trunk muscle			
TAMA (cm^2^)	86.1 (79.6–94.6)	58.8 (55.1–65.1)	0.001
SMA (cm^2^)	77.0 (67.9–83.9)	47.6 (39.8–57.2)	0.001
SMA/BMI index	3.69 (3.3–4.0)	2.7 (1.9–3.3)	0.001
Prevalence of sarcopenia (SMA/BMI <3.46), n (%)	n = 2 (11.8)	n = 12 (85.7)	0.001
IMAT/TAMA index	14.5 (11.3–18.1)	21.0 (14.1–25.6)	0.009
LAMA/TAMA index	40.4 (37.5–46.9)	48.3 (44.7–51.9)	0.006
NAMA/TAMA index	43.1 (37.4–51.0)	32.3 (26.9–33.7)	0.001
Prevalence of myosteatosis (NAMA/TAMA <65.1), n (%)	n = 17 (100)	n = 14 (100)	n/a
Individual trunk muscle			
Rectus abdominis area (TAMA, cm^2^)	6.1 (5.4–7.0)	6.0 (3.8–6.7)	0.549
IMAT/TAMA index	11.7 (10.2–21.5)	11.4 (5.3–24.9)	0.593
LAMA/TAMA index	45.6 (36.6–55.2)	51.5 (28.3–57.1)	0.941
NAMA/TAMA index	34.9 (29.1–53.9)	30.7 (17.5–64.7)	0.692
Abdominal oblique muscles group area (TAMA, cm^2^)	28.7 (23.2–33.9)	16.9 (14.0–18.7)	0.001
IMAT/TAMA index	9.8 (7.8–12.5)	13.8 (8.9–19.0)	0.098
LAMA/TAMA index	40.4 (38.2–47.0)	47.6 (44.0–54.9)	0.035
NAMA/TAMA index	47.4 (42.3–53.7)	37.1 (31.8–41.2)	0.009
Psoas major area (TAMA, cm^2^)	13.0 (11.3–16.2)	8.8 (6.5–12.7)	0.012
IMAT/TAMA index	10.4 (6.5–12.8)	10.7 (9.4–12.4)	0.413
LAMA/TAMA index	34.5 (32.2–37.0)	39.1 (36.5–43.7)	0.027
NAMA/TAMA index	54.3 (49.4–61.0)	49.2 (43.1–56.2)	0.047
Quadratus lumborum area (TAMA, cm^2^)	7.5 (6.7–9.4)	4.3 (3.8–5.1)	0.001
IMAT/TAMA index	16.0 (12.2–20.5)	20.3 (16.8–26.8)	0.048
LAMA/TAMA index	38.6 (37.1–49.0)	55.6 (51.9–60.6)	0.001
NAMA/TAMA index	38.8 (33.6–47.1)	21.8 (15.7–27.8)	0.001
Multifidus area (TAMA, cm^2^)	9.1 (8.4–10.8)	5.5 (4.2–6.7)	0.001
IMAT/TAMA index	20.2 (14.1–27.9)	25.4 (20.8–34.5)	0.119
LAMA/TAMA index	46.9 (40.0–49.9)	50.2 (47.0–54.6)	0.027
NAMA/TAMA index	31.3 (27.6–41.8)	19.6 (15.0–23.1)	0.008
Erector spinae area (TAMA, cm^2^)	21.0 (16.2–25.7)	17.4 (16.1–19.9)	0.143
IMAT/TAMA index	21.6 (13.4–24.9)	31.4 (18.2–39.7)	0.006
LAMA/TAMA index	42.6 (36.1–52.9)	49.9 (43.0–55.6)	0.178
NAMA/TAMA index	32.3 (28.4–45.9)	16.3 (14.4–22.7)	0.001

Values are presented as median (interquartile range). p-Values were calculated using the Mann–Whitney U test. A p-value <0.05 was considered statistically significant. All participants met the criteria for myosteatosis; therefore, group comparison was not performed.

n, number; TAMA, total attenuation muscle area; SMA, skeletal muscle area; BMI, body mass index; IMAT, intramuscular adipose tissue; LAMA, low attenuation muscle area; NAMA, normal attenuation muscle area; NAMA/TAMA index = (NAMA/TAMA) × 100; LAMA/TAMA index = (LAMA/TAMA) × 100; IMAT/TAMA index = (IMAT/TAMA) × 100. n/a, not applicable

For the model including the NAMA/TAMA index, the quadratus lumborum and BMI were included as explanatory variables. ORs represent the change in odds per 1% increase in each muscle composition index in the logistic regression models. The estimated ORs were 0.72 (95% CI: 0.55–0.94 p = 0.014) for the quadratus lumborum and 0.50 (95% CI: 0.27–0.93, p = 0.029) for BMI.

For the model including the LAMA/TAMA index, only the quadratus lumborum was included as an explanatory variable due to the limited number of events and to maintain model stability. The estimated OR for the quadratus lumborum was 1.28 (95% CI: 1.06–1.55, p = 0.010).

For the model including the IMAT/TAMA index, the erector spinae and BMI were included as explanatory variables. The estimated ORs were 1.29 (95% CI: 1.05–1.60, p = 0.017) for the erector spinae and 0.56 (95% CI: 0.36–0.86, p = 0.008) for BMI. The results were consistent in the sensitivity analysis using Firth’s bias-reduced logistic regression.

## Discussion

In this exploratory study, we investigated the association between trunk muscle composition at baseline and subsequent aspiration pneumonia within 1 year in older women with hip fracture. Women who developed aspiration pneumonia showed poorer muscle quality at baseline, characterized by greater intramuscular fat infiltration, compared with those without aspiration pneumonia. However, given the small sample size and potential confounding, these findings should be interpreted with caution and not as causal relationships.

Previous studies have reported that trunk muscle mass is more strongly associated with swallowing function than appendicular muscle mass^9)^. Furthermore, trunk muscle quality and posture have been associated with dysphagia and impaired swallowing function in older adults^19–21)^. In the regression analysis, muscle-specific associations were observed. The quadratus lumborum was associated with aspiration pneumonia in models using NAMA/TAMA and LAMA/TAMA indices, whereas the erector spinae were associated in the IMAT/TAMA model. These muscles play important roles in postural control and cough function and might be related to airway protection through postural stability and effective cough generation. Increased fat infiltration in these trunk muscles has been shown to impair muscle function^22)^ and has been associated with impaired swallowing and airway clearance, which may be related to the development of aspiration pneumonia.

In the muscle composition analysis, women without aspiration pneumonia showed lower IMAT/TAMA and LAMA/TAMA indices and higher NAMA/TAMA indices compared with those with aspiration pneumonia. In contrast, women who developed aspiration pneumonia showed higher LAMA/TAMA indices along with elevated IMAT/TAMA and reduced NAMA/TAMA indices. Increases in LAMA/TAMA and IMAT/TAMA indices are considered markers of greater intramuscular fat infiltration. In a large study of healthy Asian adults, the mean NAMA/TAMA index in women was reported to be 72.4, indicating a relatively high proportion of normal-attenuation muscle tissue^18)^. That study also demonstrated age-related declines in the NAMA/TAMA index and corresponding increases in LAMA/TAMA and IMAT/TAMA indices. Furthermore, lower levels of physical activity have been associated with greater intermuscular adiposity^23)^. The observed differences in trunk muscle composition in the present study may reflect differences in physical inactivity levels, which suggest the presence of muscle-specific differences rather than uniform deterioration across trunk muscles.

In addition, women who developed aspiration pneumonia showed a higher prevalence of sarcopenia, and myosteatosis was observed in all participants based on the applied cutoff value. This may reflect the advanced age and clinical condition of the study population, as well as the high prevalence of muscle quality deterioration in patients with hip fracture. The coexistence of sarcopenia and myosteatosis may reflect more severe impairment of muscle function. Importantly, trunk muscle fat infiltration assessed at baseline might reflect overall frailty or reduced physiological reserve rather than a direct causal factor for aspiration pneumonia. Previous studies have shown that muscle loss often precedes hip fracture^24)^ and that postoperative immobilization can accelerate muscle atrophy and fat infiltration^25)^. Moreover, myosteatosis has been associated with age-related functional decline^26)^, and both immobilization and insufficient nutritional intake may exacerbate sarcopenia and myosteatosis^27)^. Overall, reduced trunk muscle quality at baseline may indicate a pre-existing susceptibility to aspiration pneumonia in patients with hip fracture. These findings suggest that trunk muscle quality may serve as a potential marker for identifying women who are more likely to develop aspiration pneumonia after hip fracture.

Although causality cannot be inferred, early assessment of trunk muscle quality at baseline may help identify patients who are more likely to develop aspiration pneumonia; however, further validation is required. Causal relationships cannot be established due to the observational design and exploratory nature of this study. Therefore, these findings should be regarded strictly as hypothesis-generating.

This study has several limitations. First, the small sample size and single-center design limit generalizability and may have resulted in overfitting. Given the low events-per-variable ratio, the regression estimates may be unstable and should be interpreted with caution. In addition, given the multiple comparisons across several muscles and indices, there is a potential risk of type I error, and some observed associations may have occurred by chance. Second, selection bias may have occurred because CT was performed based on clinical indications, potentially including patients with different clinical characteristics. Third, standardized assessments of swallowing function, a key determinant of aspiration pneumonia, were not available and therefore could not be included in the analysis. This represents a major source of residual confounding and limits the interpretability of the observed associations. Therefore, the observed associations might partly reflect unmeasured differences in swallowing function rather than a direct relationship between muscle quality and aspiration pneumonia. Although patients were clinically monitored during hospitalization by attending physicians, the presence or severity of dysphagia could not be determined. Therefore, swallowing function was not only unmeasured but also could not be accounted for as a key confounder in this study. As a result, the observed associations should be interpreted as reflecting overall vulnerability rather than a direct pathway involving swallowing dysfunction. Despite these limitations, we believe that identifying trunk muscle quality as a potential marker of overall vulnerability may still have clinical relevance, particularly for early risk stratification in settings where standardized swallowing assessments are not routinely available. Therefore, the observed associations should not be interpreted as evidence of an independent or direct effect of trunk muscle quality.

## Conclusions

This exploratory study suggests a potential association between trunk muscle fat infiltration at admission and the development of subsequent aspiration pneumonia in older women with hip fracture. Trunk muscle quality assessed at admission may reflect underlying vulnerability. However, these findings should be interpreted with caution given the exploratory design, small sample size, and potential residual confounding, and no causal relationship can be established.

## Data Availability

Due to ethical restrictions, the datasets used in this study are not publicly available.
